# Erector spinae plane block in laparoscopic colorectal surgery for reducing opioid requirement and facilitating early ambulation: a double-blind, randomized trial

**DOI:** 10.1038/s41598-023-39265-5

**Published:** 2023-07-25

**Authors:** Jin-Woo Park, Eun-Kyoung Kim, Seongjoo Park, Woong Ki Han, Jiyoun Lee, Ji Hyeon Lee, Francis Sahngun Nahm

**Affiliations:** 1 Department of Anesthesiology and Pain Medicine, Seoul National University Bundang Hospital, Seongnam, Korea; 2 Department of Anesthesiology and Pain Medicine, Seoul National University College of Medicine, Seoul, Korea; 3 Department of Anesthesiology and Pain Medicine, Korea University Guro Hospital, Seoul, Korea; 4 Daeheal Pain Clinic, Seoul, Korea

**Keywords:** Diseases, Health care, Medical research

## Abstract

Various techniques have been formulated to reduce pain and ensure early recovery after surgery, as these are major concerns among surgeons, anesthesiologists, and patients. Erector spinae plane block (ESPB), the injection of local anesthetic into the fascial plane, is a simple and novel analgesia technique widely used due to its minimal risk of complications. ESPB has been tried in various surgeries; however, no study has reported its use in colorectal surgery. This study investigated whether ESPB could promote early recovery following laparoscopic colorectal surgery (LCS) by reducing opioid consumption and pain intensity. After randomization into the ESPB or control groups, an ultrasound-guided ESPB was performed at the thoracic 10^th^–11th level with 40 mL of 0.25% bupivacaine or normal saline. The ESPB group used less fentanyl during the initial 24 h after surgery (*P* = 0.004) and experienced less pain (*P* < 0.05 at all-time points) than the control group. The time to the first ambulation and the length of hospital stay were shorter in the ESPB group than in the control group (*P* = 0.015 and *P* = 0.008, respectively). In conclusion, ESPB could promote early recovery by reducing opioid consumption and pain intensity in patients receiving LCS.

## Introduction

Surgeons, anesthesiologists, and patients have been most concerned about pain and early recovery after surgery. Various efforts have been introduced to help patients recover early after surgery, and the Enhanced Recovery After Surgery (ERAS) protocol is the most comprehensive strategy for these efforts. ERAS regimens have been increasingly used to maintain physiological function, accelerate recovery, and reduce postoperative stress after surgeries^1,2^. Effective postoperative pain control without adverse effects of analgesic medications is the essential objective of ERAS pathways^3,4^. Recent studies have focused on the multimodal approaches for opioid-sparing analgesia, which uses opioids sparingly to reduce opioid-related complications, including nausea, vomiting, sedation, and respiratory depression^1,5^.

Laparoscopic colorectal surgery (LCS) is related to reduced postoperative pain, analgesic requirements, lower morbidity, faster recovery, and shorter length of hospitalization than open surgery^6,7^. Nevertheless, many patients continue to suffer from pain after LCS, which may impede recovery or rehabilitation^8,9^. Therefore, reducing pain after surgery has been considered important for the rapid recovery of patients, and for this purpose, various regional anesthesia techniques have served as an important component of multimodal anesthesia^7,10,11^.

Among various regional anesthesia techniques, the erector spinae plane block (ESPB) is a simple and novel analgesia technique widely used for its minimal risks. By injecting local anesthetics into the fascial plane, ESPB has the similar mechanism action with the paravertebral block; local anesthetics are distributed to multiple levels of paravertebral space, providing visceral and somatic analgesia in the thoracoabdominal region^12–14^. However, in contrast to the paravertebral block which is technically challenging, time-consuming, and has a substantial risk of complications, ESPB is a fascial plane block that utilizes simple sonographic landmarks and carries a low risk of serious complications^15,16^.

Although ESPB was initially used to treat severe neuropathic pain^12^, it is now increasingly used as a perioperative analgesic method in various surgeries^17,18^. Because the erector spinae muscles extend over the cervical, thoracic, and lumbar regions, and the local anesthetics used for ESPB spread both cranially and caudally over a wide range of dermatomes ^12^, when carried out at low thoracic levels, this technique can provide analgesia to the lower abdomen, which is essential for colorectal surgeries^17^. Previous clinical studies have focused on ESPB for thoracic surgery or upper abdominal surgery, such as laparoscopic cholecystectomy^14,19–21^. Nevertheless, controlled clinical data on the effects of ESPB on the postoperative recovery of patients who underwent lower abdominal surgery are insufficient.

From these backgrounds, the current study aimed to determine the efficacy of ESPB in LCS. We hypothesized that ESPB provides significant analgesic effects, decreases postoperative opioid consumption, and facilitates postoperative recovery.

## Methods

### Study design and ethical consideration

This is a double-blind, prospective randomized controlled trial. The institutional review board of Seoul National University Bundang Hospital approved this study (No. B-1907-553-002). The study protocol was registered with the University Hospital Medical Information Network Clinical Trials Registry (http://www.umin.ac.jp; registration No. UMIN000041455; registration date: August 18, 2020). All study participants were informed of this study before surgery and written informed consents were obtained. All the methods employed in this study were performed in accordance with the guidelines stipulated in the Declaration of Helsinki. We complied with the requirements established by the Consolidated Standards of Reporting Trials (CONSORT).

### Patients

The adult patients who were scheduled for elective LCS aged over 19 years and with an American Society of Anesthesiologists (ASA) physical status of class I or II were included in this study. Patients in whom needle insertion and bupivacaine injection could cause significant complications, including those with a history of allergic reaction to local anesthetics, serum creatinine level of > 2.0 mg/dL, coagulation abnormality, platelet count of < 50,000/mm^3^, active cardiovascular or cerebrovascular disease, systemic or injection site infection, and pregnant and breastfeeding patients were excluded from this study. Moreover, patients with chronic opioid medication use were excluded because their pain sensation and analgesic requirements might be abnormal. We also excluded patients with a body mass index of < 18.5 or > 30 kg/m^2^ to maintain the consistency of procedural quality.

### Randomization and blinding

An independent researcher prepared 64 identical opaque envelopes for randomization, each containing a random number from 1 to 64. Each number in the envelope was linked to the group allocation number generated by the randomization program (Research Randomizer, www.randomizer.org). Each patient was asked to select an envelope before entering the operating room. Based on the random number, each patient was assigned to either the ESPB or the control group (allocation ratio 1:1) before surgery.

### ESPB procedures

In the operating room, an independent anesthesiologist responsible for the EBPB but not involved in the rest of the protocol performed the procedure. With a patient seated, a 5–12-MHz linear ultrasonic probe (Fujifilm Sonosite Inc., Bothell, WA, USA) was placed parasagittally lateral to the spinous process between Thoracic 10th and 11th level to identify the erector spinae (ES) muscle and the adjacent transverse process. A 22-guage needle was inserted craniocaudally in plane to the transducer through the ES muscle to position the needle-tip near the corresponding transverse process and intertransverse muscles. Thereafter, 2–3 mL of 0.9% saline was injected to determine the appropriate needle position and to detach the ES muscle from the transverse process. Following the confirmation of correct needle position and absence of intravascular puncture, 20 mL of 0.25% bupivacaine at each side (total volume: 40 mL) was injected to the patients in the ESPB group. After the injection, the same procedure was repeated on the contralateral side. For the patients in the control group, an identical procedure, except for the injection of 0.9% normal saline instead of 0.25% bupivacaine, was performed. Because the injected drug was prepared before the intervention without group information, patients and the independent anesthesiologist who performed the ESPB had no knowledge of the group designation.

### General anesthesia

After the ESPB procedure, the patient’s vital signs were monitored in the supine position using electrocardiography, non-invasive blood pressure measurement, pulse oximetry, and capnography. Then, general anesthesia induction was initiated with 1.2–1.5 mg/kg of propofol and 0.6 mg/kg of rocuronium after denitrogenation with 100% oxygen. After confirming adequate muscle relaxation with train-of-four stimuli, tracheal intubation was performed (an internal diameter 7.5 mm for male and 7.0 mm for female patients). General anesthesia was maintained with 2–3 vol% of sevoflurane and a target-controlled infusion of remifentanil to maintain hemodynamic stability. Laparoscopic surgical methods, including incision size and location and number of ports, were standardized according to the type of surgery at the Seoul National University Bundang Hospital. At the end of the surgery, neuromuscular blockade was reversed with 0.01 mg/kg of glycopyrrolate and 0.04 mg/kg of neostigmine. The patients were extubated when the train-of-four ratio exceeded 90% and spontaneous breathing was established.

### Postoperative pain management

When the surgery was completed, an intravenous patient-controlled analgesia device (PCA; ANAPLUS^®^, Ewha Biomedics, Seoul, Korea) containing 15 µg/mL of fentanyl solution was applied to each patient. The PCA was implemented under the following protocol: continuous basal infusion rate of 1 mL/hour, a bolus dose of 1 mL, and a lock-out time of 15 min. At 1, 6, 12, and 24 h after LCS, a blinded researcher assessed pain severity when coughing using a 100-mm visual analog scale (0: no pain, 100: most severe pain imaginable). When the pain score was > 30, intravenous rescue analgesics (30 mg of ketorolac, followed by 50 µg of fentanyl) were administered.

### Study outcomes

The primary outcome variable was the total amount of fentanyl required in the first 24 postoperative hours. The secondary outcome variables were the time to first ambulation and length of hospitalization after surgery. The postoperative pain score at each time point, the number of rescue analgesic treatments, and the incidence of postoperative nausea and vomiting (PONV) requiring antiemetic medication during the first 24 postoperative hours were also collected.

### Statistical analysis

The number of participants required for this trial was estimated using G*power version 3.1.9.6 (Heinrich Heine University, Düsseldorf, Germany). In the pilot study, the amount of fentanyl required during the first 24 postoperative hours in patients who received LCS was 685.5 ± 220.7 (mean ± standard deviation) µg. Assuming that the standard deviation of the two groups is the same and a 25% reduction in fentanyl consumption in the ESPB group is clinically significant, the required number of participants was calculated to be 32 per group under the condition of alpha = 0.05, beta = 0.2, and an assumed dropout rate of 10%.

NCSS 2021 Statistical Software version 21.0.4 (NCSS, LLC. Kaysville, UT, USA) was used for statistical analyses. After checking the normality of the data with the Shapiro–Wilk test, we used the Mann–Whitney U test for the comparison of the continuous variables, and the chi-square test (or Fisher’s exact test) for comparing the categorical variables. Continuous variables were expressed as median with interquartile range, while categorical variables as frequency with percentage. *P-*values less than 0.05 was considered statistically significant, and all reported *P* values were from two-sided tests.

## Results

**Figure 1:**
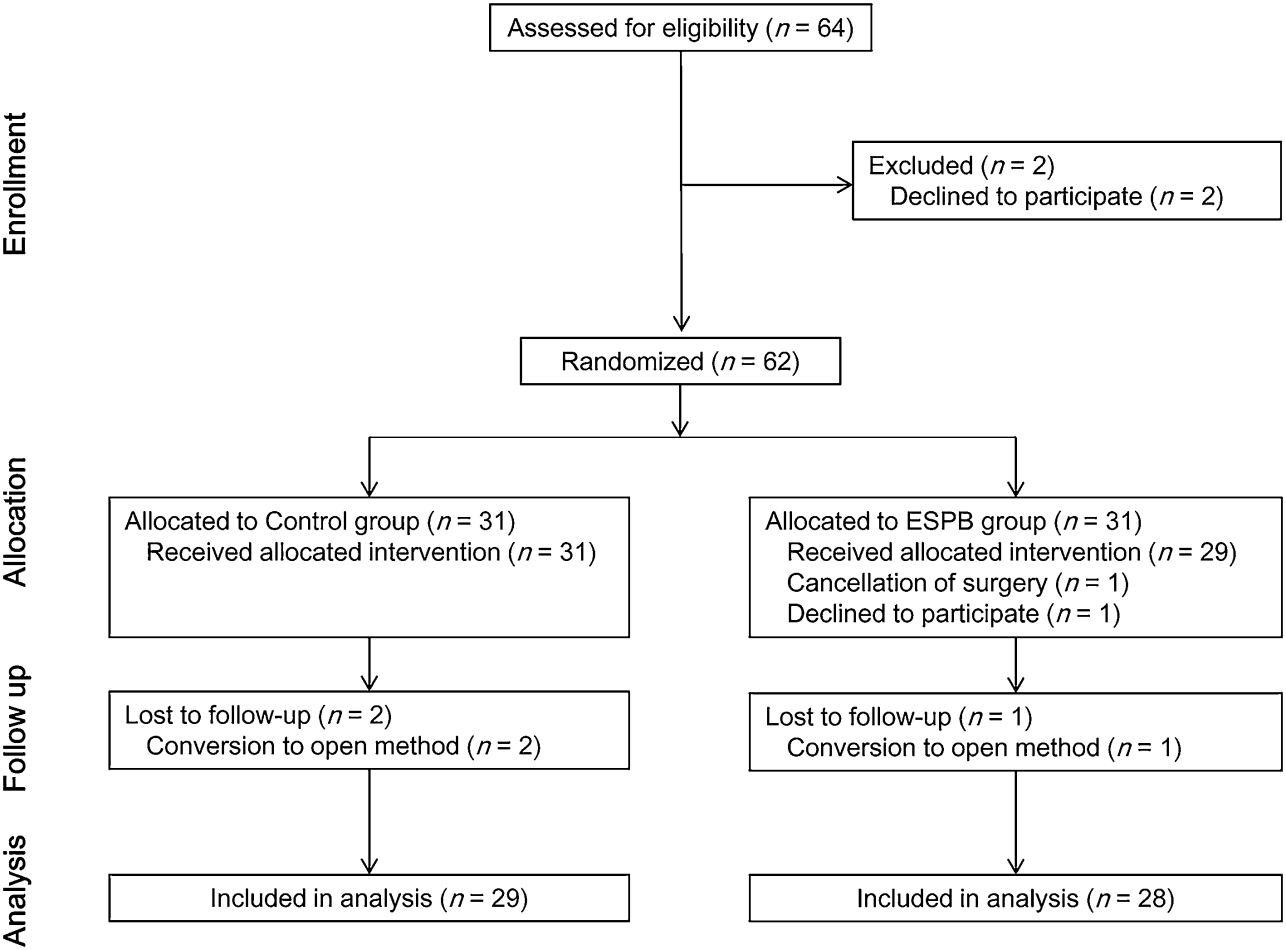
CONSORT flow diagram. ESPB, erector spinae plane block.

Among the 64 eligible patients, a total of 57 patients (n = 28 in the ESPB group, n = 29 in the control group) completed the protocol. The CONSORT diagram is illustrated in Fig. 1.

The demographic data of the patients are presented in Table 1. No significance was found in demographic and operational data between the two groups.

**Table 1:** Demographic data.

Variables	ESBP group (*n* = 28)	Control group (*n* = 29)	*P*-value
Age	60.5 (54.5–67.5)	67.0 (54.0–72.0)	0.235
Sex (male/female)	15 (54)/13 (46)	15 (52)/14 (48)	0.889
Height (cm)	160.5 (157.0–167.5)	157.4 (154.7–167.9)	0.208
Weight (kg)	61.9 (56.5–71.6)	62.2 (50.4–69.5)	0.355
BMI (kg/m^2^)	24.0 (22.3–26.0)	24.2 (21.8–25.3)	0.508
ASA class (1/2)	10(36)/18 (64)	10 (35)/19 (65)	0.922
Operation type (AR/ULAR/RH/LH)	7 (25)/16 (57)/5 (18)/0 (0)	4 (14)/16 (55)/8 (28)/1 (3)	0.474
Operation time (minutes)	147.5 (118.8–156.3)	140.0 (120.0–180.0)	0.598
Anesthesia time (minutes)	197.5 (173.8–225.0)	195.0 (175.0–240.0)	0.760
Intraoperative remifentanil dose (µg)	500.0 (337.5–662.5)	600.0 (450.0–700.0)	0.131
Laparoscopic port number	5.0 (5.0–5.0)	5.0 (5.0–5.0)	0.408

Values are presented as median (interquartile range) for continuous variables or as number (%) for categorical variables.

*AR* anterior resection, *ASA* American Society of Anesthesiologists, *BMI* body mass index, *ESPB* erector spinae plane block, *LH* left hemicolectomy, *RH* right hemicolectomy, *ULAR* ultra-low anterior resection.

**Figure 2:**
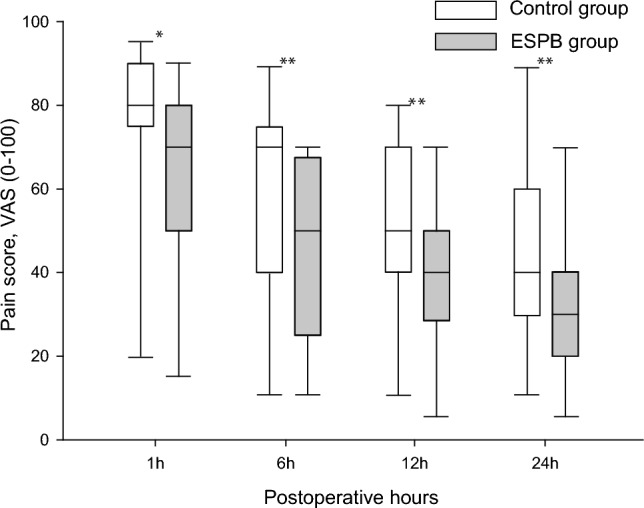
Postoperative pain scores. Box plot showing median, first, and third quartiles, and minimum and maximum values (whiskers). *p < 0.01; **p < 0.05, compared with the control. *ESPB* erector spinae plane block, *VAS* visual analog scale.

The total fentanyl requirement during the first 24 postoperative hours was significantly less in the ESPB group than that in the control group (700.0 [530.0–840.0] µg versus 530.0 [470.0–591.3] µg, *P* = 0.004; Table 2). Also, the ESPB group reported less pain than the control group at each time point (*P* < 0.05, Fig. 2).

**Table 2:** Postoperative analgesic and recovery profiles.

	ESPB group (*n* = 28)	Control group (*n* = 29)	*P*-value
Total fentanyl consumption (µg)	530.0 (470.0–591.3)	700.0 (530.0–840.0)	0.004
Nausea and vomiting (n)	4 (14.3)	8 (27.6)	0.218
Number of rescue analgesic treatments	2.0 (1.0–2.0)	2.0 (2.0–3.0)	0.098
Time to first ambulation (days)	1.0 (1.0–1.0)	1.0 (1.0–2.0)	0.015
Length of hospitalization after operation (days)	6.0 (5.0–6.0)	7.0 (6.0–8.0)	0.008

Values are presented as median (interquartile range) for continuous variables or as number (%) for categorical variables.

*ESPB* erector spinae plane block.

The incidence of PONV and the number of rescue analgesic treatments were not significantly different between the two groups (*P* = 0.218 and *P* = 0.098, respectively; Table 2). Time to first ambulation was significantly shorter in the ESPB group than in the control group (1 [1–1] day versus 1 [1–2] day, *P* = 0.015; Table 2). Length of hospitalization following surgery was significantly shorter in the ESPB group than in the control group (6 [5–6] days versus 7 [6–8] days, *P* = 0.008; Table 2). No patients complained of local anesthetic toxicity or procedure-related complications.

## Discussion

This study demonstrated that ultrasound-guided bilateral ESPB reduced fentanyl requirement and postoperative pain in the patients receiving LCS. Moreover, ESPB facilitated patient ambulation and reduced hospital stay. These results suggest that ESPB can improve the quality of postoperative recovery. This is the first randomized controlled trial to investigate the effectiveness of ESPB in terms of accelerated recovery and postoperative analgesia after LCS.

The most important finding in this study was the significant decrease in the postoperative opioid requirement. Opioid-sparing is one of the most essential therapeutic strategies of ERAS pathways. Increased perioperative opioid consumption may lead to opioid-related complications (e.g., nausea and vomiting) and long-term opioid dependency, which is significantly associated with the global opioid crisis^1,3,22^. The incidence of PONV, which is the most common side effect of opioid use, also showed no significant difference in this study. This finding may be attributed to the use of the basal opioid infusion during postoperative intravenous PCA; there could have been a significant amount of opioid consumption in both groups in this study. The total amount of remifentanil used during the surgery tended to be lower in the ESPB group than in the control group; however, the difference was not statistically significant (Table 1). This might have been due to the power analysis performed, where the primary outcome variable used for the sample size calculation was not intra-operative opioid use, but the amount of fentanyl required during the first 24 postoperative hours. Moreover, we did not regulate remifentanil infusion based on the specific hemodynamic target during surgery, which might have affected the result.

Early ambulation after colorectal surgery is essential for ERAS as it counteracts the negative physiological consequences of surgical stress^23^. Further, it is associated with less pulmonary complications and better functional mobility at discharge^1^, and it may reduce the length of hospitalization, thereby decreasing healthcare costs^23,24^. Prolonged bed rest may lead to thromboembolic complications and even insulin resistance^25^. Therefore, most ERAS guidelines strongly recommend early postoperative ambulation^23,26^. However, severe postoperative pain is the most common barrier preventing early mobilization after surgery. As shown in this study, optimal pain management is needed to facilitate mobilization after surgery^23,26^. Thus, ESPB can be considered an effective ERAS component for LCS.

Ultrasound-guided ESPB in our protocol significantly reduced the postoperative pain score at every time point. In colorectal surgery, pain intensity during the first 24 after surgery is significantly correlated with the occurrence of chronic pain three to six months after surgery^27^. Since ESPB decreased pain in the acute phase, it may reduce the likelihood of developing chronic persistent pain after LCS. Further research is needed to confirm the long-term effect of ESPB after surgery.

The analgesic effect of ESPB on visceral pain remains controversial; however, several recent clinical studies have provided supporting evidence for visceral pain relief by ESPB^13,28^. Cadaveric studies also found that local anesthetic spread resulted in blockade of rami communicants as well as ventral and dorsal rami, thereby demonstrating the potential mechanism of visceral blockade^12,29^. Thus, with respect to the postoperative period of colorectal surgery, ESPB may be superior to other regional blocks that merely provide somatic analgesia^30,31^.

Other fascial plane blocks are also performed to reduce postoperative pain in colorectal surgery. One such plane block is the transversus abdominis plane block, a simple and popular anesthetic method for abdominal surgery^10,32^. However, it may be insufficient in some surgeries as it only addresses somatic pain^14,33^. Another such plane block is quadratus lumborum block, which covers the thoracolumbar fascia and the paravertebral space and is considered an effective plane block for colorectal surgery as it relieves visceral pain as well as somatic pain^10,34^. However, quadratus lumborum block is relatively invasive, technically challenging to perform, and is associated with risk of hypotension or motor weakness^16,35,36^. EPB, on the other hand, is a relatively simple procedure with a minimal risk of complications because the target is easily visualized and vulnerable structures are far from the target^12,16,18,37^. In our study, there was no case of complication and no case where the procedure was difficult to perform.

This study may have some potential drawbacks. First, we did not determine the sensory blockade after ESPB. Determining whether the skin sensations are blocked may reveal group allocation to the physicians and patients, thereby breaking the blinding. Instead, we visually observed by ultrasound that the local anesthetics were well distributed in the target area, thereby confirming that ESPB was adequately performed. Second, the study did not include other regional blocking techniques to compare their analgesic effects. Instead, because patients in the control group received a sham block, the differences in fentanyl consumption and postoperative pain between the two groups can reflect the pain-alleviating effect of pure ESPB itself. Further research is required to explore and compare the analgesic effects of different regional anesthetic procedures (including ESPB) in colorectal surgery. Finally, the current study was performed in a single university-affiliated hospital; consequently, this may limit the generalization of the results. Furthermore, our hospital does not have an active multimodal approach to reducing perioperative opioid use. Thus, clinical data on the effects of ESPB in various perioperative settings should be investigated.

## Conclusion

In conclusion, ultrasound-guided ESPB significantly decreased fentanyl requirement and pain after LCS. This procedure also facilitated early ambulation and shortened the length of hospitalization in patients who underwent LCS. Our findings suggest that ESPB could be a powerful approach for ERAS protocols.

## Data Availability

The datasets supporting the findings of this study are available from the corresponding author upon reasonable request.
